# Education: the key to successful hip preservation

**DOI:** 10.1093/jhps/hnw011

**Published:** 2016-03-25

**Authors:** Richard (Ricky) Villar

**Affiliations:** Editor-in-Chief rnv1000@aol.com

I had a dilemma the other day. A good colleague came to me and asked whether it was essential for a surgeon to have undertaken a hip arthroscopy fellowship before practising independently as a hip arthroscopic surgeon. Instinctively I said ‘Yes’, then I said ‘No’ and shortly thereafter hesitated and said I would report back with my ponderings. Each of us knows that not all surgeons are the same and that some procedures are within our comfort zone while others lie without. Each of us definitely knows the surgeon to whom we would offer ourselves if it came to needing a hip arthroscopy. And that may not be the practitioner who makes the biggest noise.

So how does one get there? How do we create a system where hip arthroscopy is safely undertaken and yet available for as many willing surgeons to perform as is safe, practical and possible?

Perhaps the first step is to realise that hip arthroscopy is different. So is open hip preservation for that matter. However, hip arthroscopic surgery involves the use of imaging, the use of a guide wire and is also handicapped by something called the ‘fulcrum effect’. This describes the reduced tactile feedback that comes with having an arthroscope buried nearly to its eyepiece inside a patient. Look at a typical hip arthroscopic procedure underway and you will see very little arthroscope protruding from the patient. There is so much soft tissue enveloping the arthroscopic cannula that some surgeons find it very difficult to feel—and surgery is all about feeling—what is going on inside. A knee arthroscopy is different. There, you will find a significant length of cannula protruding and much less inside the knee. Tactile feedback is simpler. The abdominal surgeons are trying robotics to circumvent the problem; [1] in fact, the ophthalmic surgeons are doing the same, too [2]. Camera rotation is also key, as demonstrated by a laparoscopic study that looked at the ability of surgeons to tie knots at 0, 15, 45, 90 and 180-degree camera rotation [3]. The conclusion was that unintentional camera rotation should be avoided at surgery in order to eliminate one source of possible error.

The trouble is, not everyone can undertake hip arthroscopy, however hard they try. At least that is the implication of a fascinating study by Alvand et al. [4] who concluded that some individuals were unable to achieve competence in basic arthroscopic tasks despite sustained practice. Granted, this group was looking at the shoulder and the knee, not the hip. However, there is no reason to suppose the hip should be any different.

In terms of the number of procedures required before competence is achieved, I have always found that a difficult recommendation. Logic would decree that the more you do the better you become. Yet it is also possible for one surgeon to undertake hundreds of operations badly while another can perform just a few, but well. I recall one hand surgeon, many years ago, who undertook no more than two hip replacements every year. The problem was, he did those two hip replacements brilliantly. For him, complications simply did not exist.

In an attempt to put a number on this issue, Jackson et al. [5], looked at simulated arthroscopic meniscal repair in a study group of 19 orthopaedic residents. They concluded that although some could reach a learning plateau after performing 12 procedures, others required a further nine. However, residents also did not lose their acquired skill if they had a 6-month break from the procedure altogether. Important, perhaps, for those of us who might intend taking an arthroscopic sabbatical. Meanwhile Konan, Rhee and Haddad [6] tried to be more specific and felt the learning curve was approximately 30 cases. They looked at the first 100 hip arthroscopic procedures undertaken by a single surgeon and found there to be an overall decrease in operating time over these 100 procedures, with a decrease in complications from the first 30 cases to the remaining 70. They should be commended for writing this as not many of us would bare our chests this way in public. Personally, I reckon it took me plenty more than 30 cases to become competent at hip arthroscopic surgery and even today I can occasionally find myself stuck.

So on this basis, how important is a Fellowship? It seems that surgical skill is variable and we are not all equivalently brilliant. By inference there will be some for whom a Fellowship is critical, some for whom it is a bonus and some for whom it will be completely unnecessary. My conclusion? To make a regulation that Fellowships are essential for hip arthroscopic surgery, especially when there are so many excellent training courses in existence, is perhaps one step too far. Of course, one of the reasons JHPS does encourage correspondence is so that those of you who disagree with me are at liberty to resort to the written word and explain why.

Turning to JHPS itself, I have to say that it seems simply to be getting better, each and every time I open my Inbox or look at the latest issue. Take our last issue, e.g. issue 2.4, was that not simply one of the best collection of hip preservation papers you have seen? I have no idea where to begin. But how about the article on heterotopic ossification after hip arthroscopy presented by Amar, Sharfman and Rath? [7] There lies a complication of surgery we would rather not see. Or, perhaps the paper by Chadaymmuri et al. [8] who compared measurement of the centre-edge angle between plain radiographs and CT scan. The finding? That CT scanning consistently overinflated the result. Certainly worth keeping in mind when you are next measuring femoral head coverage. Do not trust that plain radiographs and CT scanning will agree.

Meanwhile looking at this issue, issue 3.1, the first of 2016, I was especially intrigued by Sansone et al’s paper [9] on the outcome of hip arthroscopy in patients with mild-to-moderate osteoarthritis. This was a prospective study over 2 years, which found patients had a statistically significant chance of improvement after hip arthroscopy. This flies in the face of accepted orthopaedic dogma and is pleasing to see as I am sure a number of us undertake hip arthroscopic surgery in the presence of osteoarthritis, are careful not to promise the patient too much and yet still manage to see an improvement after our surgery. Arthroscopy and osteoarthritis are not, in my view, always incompatible bed fellows although patient expectations are key.

So once again, on behalf of JHPS, this journal, our journal, my sincerest thanks for the support you have all shown and continue to demonstrate. In almost record time, between us we have somehow put JHPS on the global academic map. Brilliant.

My very best wishes to you all.
